# Retrospective study of hypofractionated stereotactic radiotherapy combined with whole brain radiotherapy for patients with brain metastases

**DOI:** 10.1186/s13014-022-02096-1

**Published:** 2022-07-26

**Authors:** Xue-Yi Xie, Hong-Hua Peng, Xi Zhang, Yu-Liang Pan, Zhen Zhang, Pei-Guo Cao

**Affiliations:** Department of Oncology, The Third Xiangya Hospital of Central South University, Changsha, People’s Republic of China

**Keywords:** Brain metastases, Whole brain radiotherapy, Hypofractionated stereotactic radiotherapy, Efficacy, Safety

## Abstract

**Background and purpose:**

To evaluate the clinical outcomes of hypofractionated stereotactic radiotherapy (HFSRT) combined with whole brain radiotherapy (WBRT) in patients with brain metastases (BMs).

**Materials and methods:**

From May 2018 to July 2020, 50 patients (111 lesions) received HFSRT (18 Gy/3F) + WBRT (40 Gy/20F). The RECIST 1.1 and RANO-BM criteria were used to evaluate treatment efficacy. Five prognostic indexes (RPA, GPA, SIR, BS-BM, and GGS) were applied. The primary endpoint was intracranial local control (iLC). Secondary endpoints were overall survival (OS) and the safety of treatment.

**Results:**

Intracranial objective response rates (iORR) using the RECIST 1.1 and RANO-BM criteria were 62.1% and 58.6%, respectively. The iLC rate was 93.1%, the 6- and 12-month iLC rates were 90.8% and 57.4%, respectively. The median intracranial progression-free survival (iPFS) was not reached (range 0–23 months). The 6-, 12-, and 24-month OS rates were 74.2%, 58.2%, and 22.9%, respectively. The KPS score showed statistical significance in univariate analysis of survival. The 6, 12, and 24 month OS rates for patients with KPS ≥ 70 were 83.8%, 70.5%, and 29.7%, respectively. The median survival time (MST) for all patients and for patients with KPS ≥ 70 were 13.6 and 16.5 months, respectively. Sex, KPS score, and gross tumor volume were significant factors in the multivariate analysis of survival. OS was significantly associated with RPA, SIR, BS-BM, and GGS classes. No acute toxicities of grade 3 or higher were noted.

**Conclusion:**

HFSRT combined with WBRT is a safe and effective local treatment modality for BM patients.

## Introduction

The development of brain metastases (BMs) is a common complication in patients with advanced malignant tumor, 10–40% of patients with solid tumors will develop BMs over their clinical course [1]. The treatment of BMs mainly includes systemic and local treatment, but conventional systemic therapy often achieves unreliable penetration through the blood–brain barrier (BBB). The current standard local treatment for BMs, consisting of a multimodal approach including surgery, stereotactic radiosurgery (SRS), hypofractionated stereotactic radiotherapy (HFSRT) and whole brain radiotherapy (WBRT) [2, 3]. With the development of radiotherapy, the role of surgery in local treatment is gradually reduced, and the radiotherapy is more recommended as the initial local treatment for BMs patients [4].

Recently, HFSRT has been increasingly used to treat BMs [5, 6]. Some reports describing the treatment results of HFSRT versus those of SRS, indicated that HFSRT had similar efficacy and lower toxicity compared to SRS, especially for large lesions [7, 8]. In addition, the ideal dose-fractionation scheme of HFSRT is controversial [9, 10]. In many previous studies, patients with a poor Karnofsky performance status (KPS) were considered ineligible for HFSRT [7, 11], but it remains unclear that whether patients with a poor KPS (defined as KPS < 70) due to BMs are ineligible for HFSRT [12]. As for WBRT, despite the potential risk of WBRT damage to neurocognitive function, studies have shown that it can significantly reduce intracranial tumor recurrence, and its effect on neurocognitive function is much less than that of patients with neurological impairment or loss caused by tumor progression [13, 14]. And it was observed in our clinical experience that the intracranial recurrence rate of patients with BMs without WBRT treatment was higher; thus, WBRT might be reserved in selected patients.

Conversely, the response and progression criteria used in clinical trials on BMs are disparate [15]. The Response Evaluation Criteria in Solid Tumors (RECIST) criteria generalizes the complexity of tumor geometry to a linear dimension, disregarding the indication that lesion enlargement after treatment includes both radiation necrosis and disease progression [16]. The Response Assessment in Neuro-Oncology Brain Metastases (RANO-BM) standard is currently a comparatively comprehensive evaluation standard, but it requires confirmation by more clinical studies [15].

It seems noteworthy that the prognostic indexes (PIs) that are widely used in clinical practice include the recursive partitioning analysis (RPA) class [17] and the graded prognostic assessment (GPA) score [18]. With the gradual increase in the application of local treatments, some scholars have begun to study prognostic scoring systems that are based on stereotactic radiotherapy (SRT), such as the score index for radiosurgery (SIR) [19], the basic score for BM (BS-BM) [20], and the golden grading system (GGS) [21]. It has not been established which PI is most appropriate for patients with BMs who are receiving HFSRT + WBRT.

This study aimed to evaluate the efficacy and toxicity of HFSRT + WBRT in patients with BMs. We also sought to determine which of the five PIs was most suitable in our study, and we compared the accuracies of RECIST 1.1 and RANO-BM standards for efficacy evaluation.

## Materials and methods

From May 2018 to July 2020, we enrolled patients with BMs who underwent HFSRT + WBRT in our hospital. The inclusion criteria were: (1) patients with histologically confirmed malignancies who had 1–10 intraparenchymal brain metastases and (2) patients aged ranged from 18 to 80 years. The exclusion criteria were: (1) patients with a history of WBRT, SRT, or any other form of intracranial irradiation, (2) patients who received follow-up for < 1 month.

The study was approved by the institutional review board of our hospital; no patient consent was required owing to the retrospective study design.

**Fig. 1:**
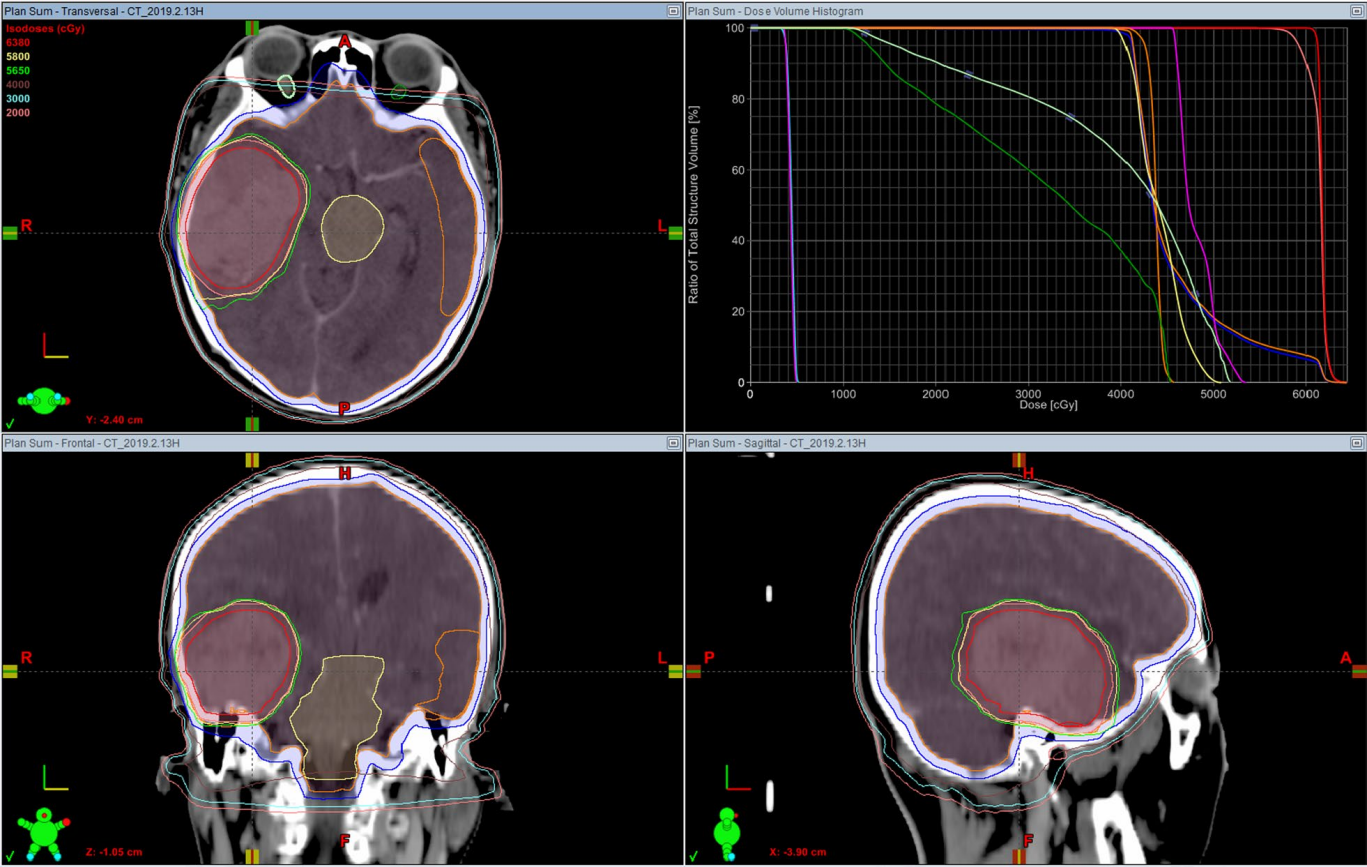
Target volumes of radiotherapy for brain metastases

HFSRT was delivered by Linear Accelerator (Varian, USA) at our institution. Patients were immobilized in a supine position with a thermoplastic head mask fixation system (Klarity, Guangzhou, China), simulating a high-resolution thin slice (1.2 mm) computed tomography (CT). The target volumes and organs at risk were contoured using the Eclipse version 11.0 (Varian, USA) treatment planning system. Gross tumor volume (GTV) of HFSRT was defined based on the enhanced volume that was detected during MRI T1-weighted contrast-enhanced sequencing. A 3.0 mm three-dimensional expansion was applied to the GTV to create the planning gross tumor volume. Patients were treated with  TrueBeam Linear Accelerator (Varian, USA). Typical target volumes for BMs are shown in Fig. 1. The prescription dose of HFSRT was 18 Gy in 3 fractions, and the prescription dose of WBRT was 40 Gy (2 Gy per fraction) for whole brain planning target volume. The median time between diagnosis of BMs and HFSRT was 16.5 days. Patients received HFSRT daily, followed by WBRT, from Monday to Friday; no treatment was administered over the weekends.

Follow-up of patients included the collection of clinical and head imaging data. Enhanced MRI or CT evaluations were scheduled for before treatment, 1 month after HFSRT, and every 3 months subsequently, until treatment failure or death. The assignment of central nervous system response was independent of systemic disease response. The treatment response of BMs was classified as complete response (CR), partial response (PR), stable disease (SD), and progressive disease (PD), according to the RECIST (version 1.1) [22] and RANO-BM criteria [15]. Acute toxicity was classified according to the Common Terminology Criteria for Adverse Events (CTCAE) version 4.0. Radionecrosis was diagnosed based on MRI changes (including diffusion-weighted imaging, perfusion-weighted imaging, arterial spin labeling, and MR spectroscopy) consistent with necrosis (central hypodensity and peripheral enhancement on T1-weighted post-contrast imaging, with edema on T2-weighted sequences) in the setting of new neurologic symptoms or a new steroid requirement [23].

The primary endpoint was intracranial local control (iLC). The iLC was defined as any intracranial lesion that did not meet the criteria for PD, in the absence of new intracranial lesions. The intracranial objective response rate (iORR) was defined as proportion of patients who obtained intracranial CR and PR. The intracranial progression-free survival (iPFS) was defined as time between the start of HFSRT and intracranial PD. The secondary endpoint were OS and the safety of treatment. The OS was defined as the time from HFSRT to death or loss to follow-up.

Numerical variables are expressed as median, interquartile range (IQR), and range. Qualitative data are expressed as frequency and percentage. The Kaplan–Meier method was used to construct survival curves and determine the median survival time (MST). The Cox proportional hazards model was used in univariate and multivariate analyses. The confidence interval (CI) was designated 95%.

## Result

### Patient and lesion characteristics

Between May 2018 and July 2020, we enrolled 54 patients who had undergone HFSRT + WBRT. Of these patients, 2 patients were followed up for < 1 month, 1 patient had a primary tumor with no pathological diagnosis, and 1 patient was 83 years old. These four patients were excluded from the analysis. The final analysis included 50 patients with a total of 111 lesions. The median time interval between primary tumor diagnosis and the manifestation of BMs was 7.8 months (range 0–83.1 months). Metachronous BMs were considered present when the time between primary tumor diagnosis and the occurrence of intracranial metastasis exceeded 3 months [24]. The median age of all patients was 56 years (range 31–77). The median number of target BMs was 2 (range 1–6). The median diameter of lesions was 2.30 cm (range 0.60–5.50 cm), and the median size of GTV was 8.56 cm^3^ (range 0.87–88.28 cm^3^).

### Intracranial efficacy

Patients who did not undergo CT/MRI in our institution before and after HFSRT were excluded. Eventually, 29 patients were included in intracranial efficacy analysis. The number of BMs in the patients varied between 1 and 6, and the median GTV size was 7.24 cm^3^ (range 0.87–88.28 cm^3^). Only 42 target lesions could be evaluated by the RECIST 1.1 criteria because the RECIST 1.1 criteria stipulates that each organ can have a maximum of two target lesions; 48 target lesions could be evaluated by the RANO-BM criteria.

**Fig. 2:**
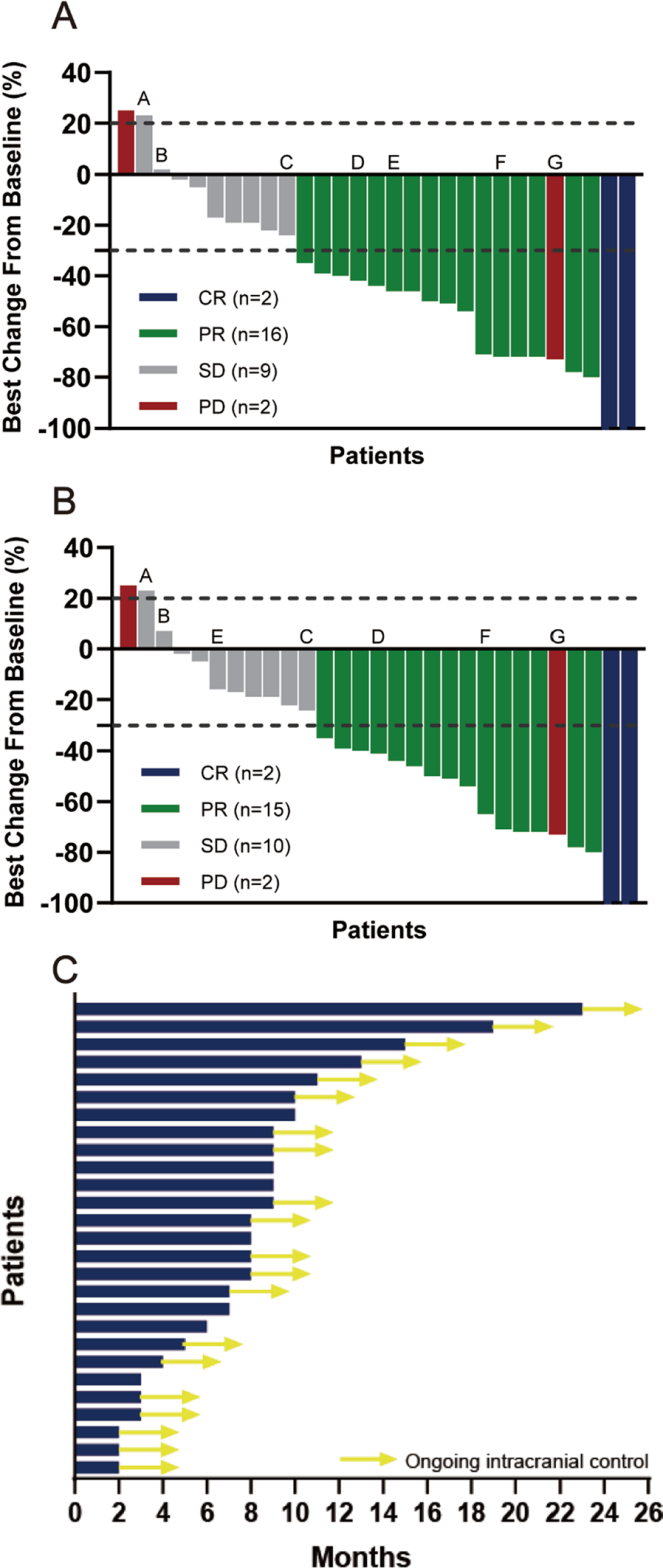
Tumor responses to hypofractionated stereotactic radiotherapy combined with whole brain radiotherapy. Plane **A** shows the best response of patients evaluated by the RECIST 1.1 criteria. Plane **B** shows the best response of patients evaluated by the RANO-BM criteria. The bars indicate the best percent change in target tumor burden from baseline. Letter A denotes a patient with a > 20% increase in the sum of the longest diameters of target lesions, but the absolute value of the diameter increase was only 3 mm; therefore, the efficacy assessment remains as SD. Letter C denotes a patient who was suspected to have a new intracranial lesion, but the lesion was < 10 mm in diameter on consecutive reexaminations and were assessed as SD. The letters B, D, and F indicate three patients with different numbers of target lesions in the two evaluation criteria, who had the same evaluation results. The letter E indicates a patient with different numbers of target lesions in the two evaluation criteria, which resulted in different evaluation results. The letter G denotes a patient with reduced target lesions but multiple new intracranial lesions. Plane **C** shows the intracranial progression-free survival of the 27 patients.

We recorded the greatest change from baseline in the longest diameter of target lesions and evaluated treatment efficacy based on CR, PR, SD, and PD in the RECIST 1.1 (Fig. 2a) and RANO-BM criteria (Fig. 2b). The iORR of the 29 patients using the RECIST 1.1 and RANO-BM criteria was 62.1% and 58.6%, respectively. The iLC rate was 93.1% for both criteria, with the 6- and 12-month iLC rate of 90.8% and 57.4%, respectively. Two patients with efficacy evaluations of PD, one with target lesion progression and the other with distant intracranial progression. The median iPFS was not reached (range 0–23 months). At the time of data cutoff, 20 of the 27 intracranial controls (74.1%) had ongoing intracranial control (Fig. 2c). Of the 7 patients who progressed during follow-up, 2 had target lesion progression and 5 had distant intracranial progression.

### Analysis of survival

**Fig. 3:**
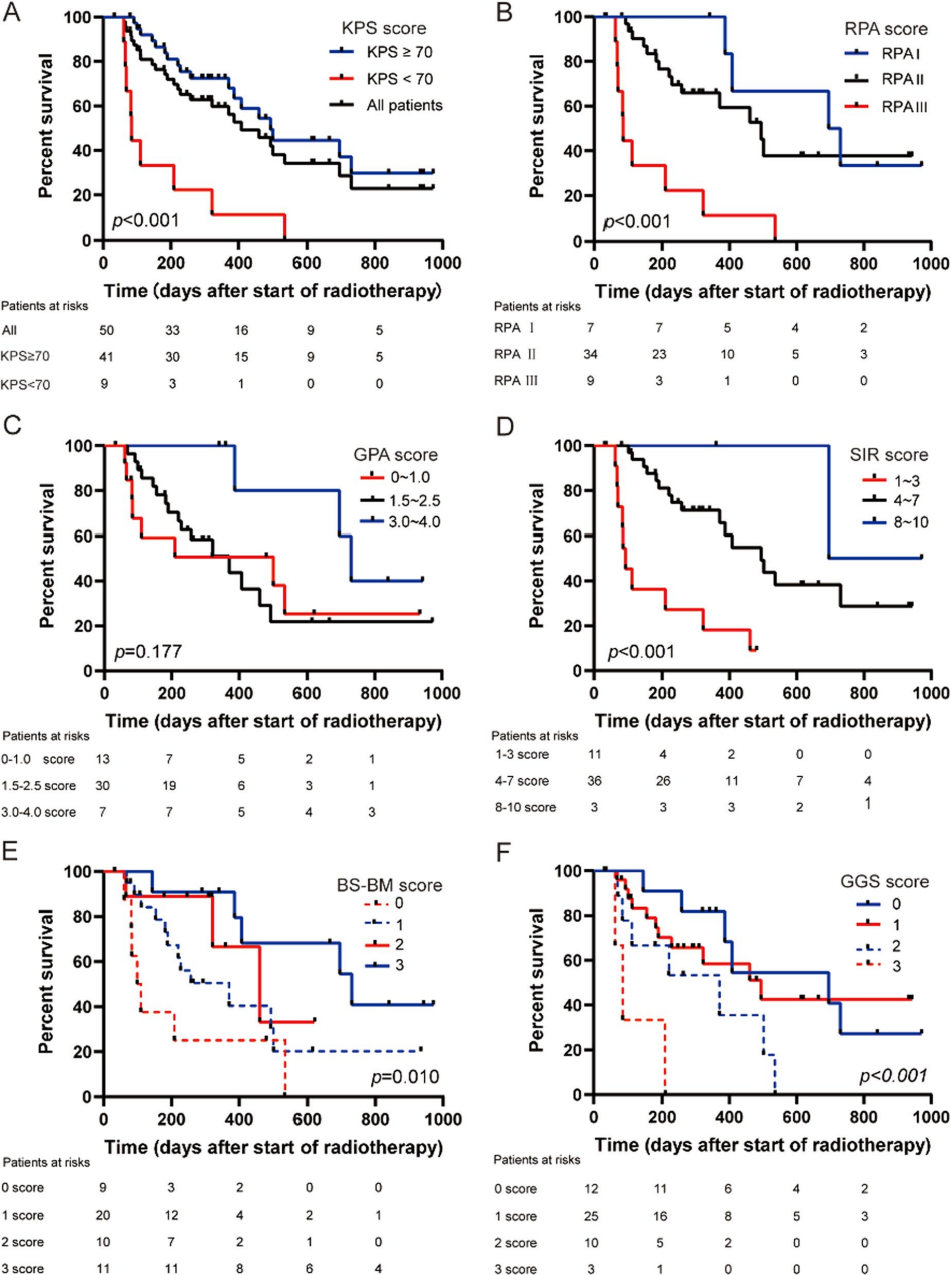
Kaplan–Meier curve of overall survival in patients with brain metastases. **A** Kaplan–Meier curve of overall survival in patients with different KPS scores. **B** Overall survival for different RPA scores (n = 50); **C** Overall survival for different GPA scores (n = 50); **D** Overall survival for different SIR scores (n = 50); **E** Overall survival for different BS-BM scores (n = 50); **F** Overall survival for different GGS scores (n = 50)

At the time of analysis (January 2021), 27 patients (54.0%) had died. Three patients from radiologically confirmed intracranial progression, 14 from extracranial progression, 7 from unspecified progression or unknown causes, and 3 from clinical complications related to systemic disease (pulmonary infection, multiorgan failure and hemorrhage of digestive tract). The median follow-up time was 9.3 months (IQR, 3.7–16.2) for 50 patients. The MST was 13.6 months (95% CI 9.0–18.2 months) and the 6-, 12-, and 24-month actuarial OS rates were 74.2%, 58.2%, and 22.9%, respectively (Fig. 3a).

**Fig. 4:**
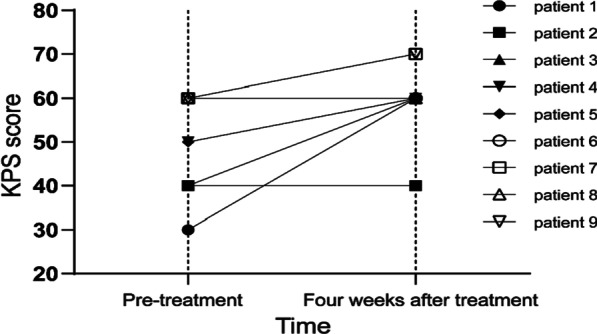
Changes in the Karnofsky performance status (KPS) score 4 weeks after hypofractionated stereotactic radiotherapy (HFSRT) in nine patients with KPS score < 70 before treatment

Eight potential prognostic variables were included in univariate analyses. As shown in Table 1, only KPS score (HR 5.455; 95% CI 2.384–12.482; *p* < 0.001) was statistically significant. The median OS was 2.8 months (range 2.0–17.9) for patients with KPS < 70. For patients with KPS ≥ 70, the MST was 16.5 months (95% CI 12.2–20.7 months), and the 6-, 12-, and 24-month actuarial OS rates were 83.8%, 70.5%, and 29.7%, respectively (Fig. 4a). Sex (HR 4.546; 95% CI 1.601–12.907; *p* = 0.004), KPS score (HR 10.754; 95% CI 3.911–29.571; *p* < 0.001), and GTV size (HR 0.288; 95% CI 0.118–0.698; *p* = 0.006) were significant factors in the multivariate analysis (Table 1).

**Table 1:** Median survival time according to potential factors and the results of univariate and multivariate analyses

Variables	N (%)	MST (months)	*P* value	
			Univariate	Multivariate
*Sex*			0.146	0.004
Male	32 (64.0)	12.4		
Female	18 (36.0)	23.2		
*Age*			0.532	0.630
< 60	27 (54.0)	12.9		
≥ 60	23 (46.0)	15.4		
*KPS*			< 0.001	< 0.001
≥ 70	41 (82.0)	16.5		
< 70	9 (18.0)	2.8		
*Primary tumor location*			0.619	–
Lung cancer	39 (78.0)	13.6		
Others	11 (22.0)	12.4		
*Extracranial metastases*			0.205	0.124
Existent	31 (62.0)	12.4		
None	19 (38.0)	15.4		
*Contemporaneous BM*			0.615	–
Yes	19 (38.0)	12.9		
No	31 (62.0)	13.6		
*Number of BMs*			0.872	–
Single	19 (38.0)	12.9		
Multiple	31 (62.0)	15.4		
*GTV (cm*^*3*^*)*			0.092	0.006
< 10.0	29 (58.0)	16.7		
≥ 10.0	21 (42.0)	12.9		

MST, median survival time; BMs, brain metastases; GTV, gross tumor volume

Survival analysis was performed for lung adenocarcinoma patients who were treated with (15 patients) or without (10 patients) targeted therapy, and the MST was 17.9 months (95% CI 8.1–27.6 months) and 12.9 months (95% CI 6.2–19.6 months), respectively (HR 0.460; 95% CI 0.166–1.278; *p* = 0.136).

### The five PIs

Patients were classified into 3 or 4 classes, according to the corresponding grading requirements of PRA, GPA, SIR, BS-BM, and GGS. Patient characteristics and survival analysis for each PI are listed in Table 2. Survival curves are shown in Fig. 3. OS was significantly associated with the RPA, SIR, BS-BM, and GGS classes. There was no statistical significance between the GPA classes.

**Table 2:** Patient characteristics and survival analysis for the five prognostic indices

PI	Grade	No. of patients (%)	MST (months)	*p*
RPA				< 0.001
	Class I	7 (14.0)	23.8	
	Class II	34 (68.0)	16.5	
	Class III	9 (18.0)	2.8	
GPA				0.177
	0–1.0	13 (26.0)	16.7	
	1.5–2.5	30 (60.0)	12.4	
	3.0–4.0	7 (14.0)	24.3	
SIR				< 0.001
	1–3	11 (22.0)	3.0	
	4–7	36 (72.0)	16.5	
	8–10	3 (6.0)	27.8	
BS-BM				0.010
	0	9 (18.0)	3.5	
	1	20 (40.0)	12.4	
	2	10 (20.0)	15.4	
	3	11 (22.0)	24.3	
GGS				< 0.001
	0	12 (24.0)	23.2	
	1	25 (50.0)	16.5	
	2	10 (20.0)	12.4	
	3	3 (6.0)	2.8	

MST, median survival time; RPA, Recursive Partitioning Analysis; GPA, Graded Prognostic Assessment; SIR, Score Index for Radiosurgery; BS-BM, Basic Score for Brain Metastases; GGS, golden grading system

### BM-related symptoms

Of the 50 enrolled patients, 39 had symptoms associated with BM prior to treatment and 11 were asymptomatic at the time of BM diagnosis. These 39 patients were divided into two groups according to their KPS score. All 9 patients with KPS < 70 had BM-related symptoms before treatment, which mainly included muscle weakness, dizziness, headache, unsteady gait, numbness, blurred vision or partial blindness, and symptomatic epilepsy. These symptoms seriously affected the quality of life of the patients. Most of the patients showed significant improvement in dizziness, headache, and limb weakness, but no significant improvement in vision loss was associated with BMs. One patient with symptomatic epilepsy before treatment was poorly managed with sodium valproate, with no recurrence of seizures after treatment. For patients with KPS score < 70, symptoms related to BMs before and after treatment and changes in KPS score are shown in Table 3 and Fig. 4, respectively. The main symptoms in patients with KPS ≥ 70 included headache, dizziness, muscle weakness, and numbness. Symptoms improved in some patients after treatment, while some symptoms such as blurred vision did not improve significantly (Table 4).

**Table 3:** Symptoms related to brain metastases in nine patients with KPS score < 70 pre-treatment and 4 weeks after treatment

Patient	Pre-treatment		Four weeks after treatment	
	Main symptoms	KPS score	Symptoms	KPS score
1	Muscle weakness right-sided and dizziness	30	Relief of muscle weakness and dizziness	60
2	Left eye hemianopsia and Headaches	40	No change in visual acuity; relief of headaches	40
3	Symptomatic epilepsy	40	Relief of seizure	60
4	Facial spasm, glossolalia, and dizziness	50	Relief of glossolalia and dizziness	60
5	Dizziness and blurred vision	50	Relief of dizziness	60
6	Muscle weakness in the lower limb	60	Roughly as before	60
7	Numbness of the lower limb	60	Relief of numbness	70
8	Numbness of the right limb and nausea	60	Roughly as before	60
9	Dizziness and unsteady gait	60	Relief of dizziness and unsteady gait	70

**Table 4:** Symptoms related to brain metastases in patients with KPS score ≥ 70 pre-treatment and 4 weeks after treatment

Symptoms	Number of patients				
	Pre-treatment	Four weeks after treatment			
		Upturn	No change	Deterioration	New symptoms
Headache	12	11	1	0	0
Dizziness	10	5	5	0	0
Nausea	3	2	1	0	0
Vomiting	4	3	1	0	0
Tremor	1	1	0	0	0
Muscle weakness in the upper limb	3	2	1	0	0
Muscle weakness in the lower limb	5	3	2	0	0
Motor weakness	4	3	1	0	0
Numbness	6	4	2	0	0
Unsteady gait	2	1	1	0	0
Glossolalia	2	1	1	0	0
Blurred vision	2	0	2	0	0
Hiccup	1	0	1	0	0

### Safety of treatment

Radiation toxicities are reported using the CTCAE (version 5.0). Regarding acute toxicity, all patients tolerated the treatment well with no ≥ grade 3 toxicities. Only grade 1 or 2 adverse events were reported. The most common side effect was fatigue. Concerning late toxicity, intra-tumoral hemorrhage complications occurred in one patient (2%) 9 months after treatment completion, but this did not progress into a neurologic degradation.

## Discussion

HFSRT + WBRT showed good results in terms of efficacy, toxicity and survival time.

Based on previous clinical experience and the results of this study, we considered that WBRT could not be ignored. Some studies suggest that SRT can be considered a standard treatment that is a less toxic alternative to SRT + WBRT [25]. However, WBRT reduction of brain tumor recurrence rate may translate to improved survival in patients with intracranial tumor progression; thus, it seems reasonable to suggest that the addition of WBRT may affect survival outcomes in selected patients [26, 27]. Wegner RE et al. reviewed 36 patients who were treated with HFSRT alone (24 Gy in2-5F), with 6- and 12-month LC rates of 73% and 63%, respectively [28]. Kim et al. reviewed 46 patients who were treated with HFSRT alone. Patients were randomized to receive 24, 27, or 30 Gy in 3 fractions, with 12-month LC rates of 65%, 80% and 75%, respectively [11]. The intracranial LC rate in this study was superior to the two studies mentioned above, further studies are needed to investigate the most suitable population for HFSRT + WBRT.

In our study, the HFSRT group received a smaller total dose and number of fractions than those reported in the literature. We reviewed recent studies on patients with BMs who were treated with HFSRT or HFSRT + WBRT; the most common HFSRT dose was 27 Gy/3F (range 24–41 Gy/2–6F), the OS rates ranged from 13.0 to 69.0% at 12 months, and the MST ranged from 12.2 to 16.2 months [6, 7, 11, 28–31]. Our results are also comparable to those of several studies on HFSRT for BMs in the surgical cavity. Two studies assessed the efficacy and safety of postoperative HFSRT in patients with BMs. The 12-month OS was 62% and 58%, respectively. The radiation necrosis rate was 5.1% and 8.9%, respectively [32, 33].

PI are beneficial to clinical and therapeutic decision-making. However, GPA classes were not statistically significant in our analysis. Conversely, many previous studies concluded that GPA is a reliable, simplistic, and powerful tool for predicting survival [18, 34]. The possible reasons for this difference include the following. First, GPA includes the number of metastases and does not involve metastasis volume. We noted that the number of BMs in the patients and the GTV size were not proportional. The median GTV was greater in patients with 1 BM than in patients with 2–3 BMs (9.45 and 7.25 cm^3^, respectively). Some studies concluded that brain tumor volume had a significant association with OS [35, 36]. Second, the primary tumors in our study included lung cancers, breast cancers, gastrointestinal cancers, and gynecologic tumors. There was marked heterogeneity in outcomes among patients with BMs and the differences in outcome were not only related to the diagnosis but also to diagnosis-specific prognostic factors [34, 37]. Further, the dissimilar proportions of patients within the prognostic classes could be another reason. In addition, we found that the PIs in our study were not uniformly recommended in different studies, and there was a large discrepancy between the expected and actual survival [18, 38–40].

In this study, we used two instruments for efficacy evaluation. There was no significant difference in efficacy outcomes between the two criteria; this was possibly due to: (1) the small number of patients included in the efficacy analysis, (2) of all patients treated with HFSRT, only one patient showed asynchronous changes in the intracranial lesions, which may have attenuated the difference in the number of target lesions between the two criteria, and (3) although corticosteroid use is not included in the RECIST 1.1 criteria, no patient who met the imaging criteria was ineligible for corticosteroid use, thus eliminating the potential discrepancy from this definition. The RANO-BM criteria, may provide a more comprehensive assessment of patient outcomes than the RECIST 1.1 criteria. However, in our practical application of the RANO-BM criteria we found that the criteria complicate the assessment of patients with BMs in clinical trials.

We believe the results of the present study are important for several reasons. First, we used the same HFSRT and WBRT scheme for the entire study, reduced treatment-derived differences in the analysis of treatment efficacy. Second, to our knowledge, our study involved the lowest total radiation dose and number of fractions, but we observed desirable survival outcomes. Third, we analyzed survival outcomes and symptom and KPS score improvement in patients with a poor KPS post-treatment. Our findings demonstrate that patients with KPS < 70 may not be unfavorable candidates for SRT. Lastly, to our knowledge, this is the only study that has employed both RECIST 1.1 and RANO-BM criteria to evaluate the HFSRT + WBRT efficacy for BM.

This study was limited by its retrospective design. During the radiotherapy and follow-up periods, patients also received systemic treatments, which might influence their survival and local control. In addition, we did not avoid the hippocampal regions during irradiation in WBRT. We also did not evaluate the neurocognitive function of patients, post-treatment using scores.

## Conclusions

Despite limitation mentioned above, these data demonstrated that, for patients with BMs, HFSRT combined with WBRT is a safe and effective local treatment modality. Patients with a poor KPS due to BMs can also benefit from the treatment. The response and progression criteria for patients with BMs remain to be explored in further studies.

## Data Availability

All data generated or analysed during this study are included in this published article.
